# 4025 Fall Risk Screening and Referrals to Community-Based Programs among Physical Therapy Professionals

**DOI:** 10.1017/cts.2020.391

**Published:** 2020-07-29

**Authors:** Jennifer L. Vincenzo, Tiffany Shubert, Jennifer S. Brach, Jennifer Tripken, Lori Schrodt, Jennifer C. Sidelinker, Patrice Hazan, Colleen Hergott, Kathleen Shirley, Beth Rhorer

**Affiliations:** 1 University of Arkansas Translational Research Institute; 2 Academy of Geriatric Physical Therapy; 3 University of Pittsburgh; 4 National Council on Aging; 5 Western Carolina University; 6 Genesis Healthcare; 7 GroupHab Physical Therapy; 8 Augusta University; 9 Texas Women’s Univeristy; 10 Centreville Physical Therapy

## Abstract

OBJECTIVES/GOALS:

- To describe trends in fall risk screening and referrals to community-based programs among physical therapy professionals.
- To compare fall risk screening practices to clinical practice guidelines among
- To identify gaps in fall risk screening and referrals to community-based programs among physical therapy professionals.

METHODS/STUDY POPULATION: A panel of experts between the American Physical Therapy Association (APTA) - Geriatrics, and the National Council on Aging (NCOA) developed a web-based survey to identify practices among physical therapy professionals (PTs) for fall risk screenings and community-based referrals for older adults. The web-based survey was disseminated to PTs via email, e-blasts, and social media. The survey focused on questions related to knowledge of fall risk screening tools, fall risk management for older adults, and knowledge of and referrals to community-based interventions. RESULTS/ANTICIPATED RESULTS: To date, 453 PTs representing 50 states completed the survey. The majority of PTs (50.9%) had over 20 years of experience in various settings. Eighty-three percent regularly screen older adults for fall risk. Approximately 40% conduct community-based screenings. The majority (81.3%) were somewhat to very familiar with the CDC-recommended STEADI (Stopping Elderly Accidents, Deaths, and Injuries) screening toolkit. Despite familiarity, only 32% responded to the question if they used STEADI for screening. Of those, 83.4% used the tool. The majority (73.4%) of PTs were aware that NCOA recommends evidence-based programs to address health needs of aging adults and 59.6% refer. PTs did not refer due to lack of knowledge that programs existed (21.3%) or lack of knowledge of availability (33.3%). DISCUSSION/SIGNIFICANCE OF IMPACT: Although PTs are have some familiarity with the STEADI for fall risk screening, the tool is not common in practice. PTs are lacking awareness of local evidence-based community programs to address health needs of aging adults. Educational efforts should target these knowledge gaps and provide additional resources to improve referrals.

